# Supplementary material for: Hybrid deep layered network model based on multi-scale feature extraction and deep feature optimization for acute lymphoblastic leukemia anomaly detection
Source: PeerJ Comput Sci. 2025 Sep 4;11:e3174. doi: 10.7717/peerj-cs.3174 (PMC12453709; doi:10.7717/peerj-cs.3174)
Supplement: Supplemental Information 2 [file peerj-cs-11-3174-s002.rtf]

Hybrid Deep Layered Network Model Based on Multi-scale Feature Extraction and Deep Feature Optimization for Acute Lymphoblastic Leukemia Anomaly Detection
This research uses the enrichment capabilities of hybrid deep transfer learning to accurately and effectively diagnose different conditions of ALL disease. The dataset of blood cell images is used to train and test the proposed deep learning models.
Dataset (URL/DOI):
CNMC-2019  Dataset: https://doi.org/10.7937/tcia.2019.dc64i46r

System Configuration
NVIDIA GEFORCE RTX 3080 GPU and 32 GB DDR5 RAM were used for training the deep learning architectures used in the experiments performed for ALL detection. OS, OpenCV, Numpy and Pandas libraries were used in the image pre-processing stage. Keras and Tensorflow libraries were used for training the deep learning architectures.

Preprocessing:
After completing the software requirements and dataset installation. The comprehensive preprocessing was performed on the dataset to make it compatible for the deep learning model as shown below:
STEPS INVOLVED IN DATA PROPROCESSING	
Input: Database with 2 distinct classes
·	Data Loading and statistical analysis.
·	Center clipping is applied to reduce visible noise.
·	ImageDataGenerator class is exploited to create training, validation and testing sets.
·	Preprocessed data is provided to the proposed model.	

Model Training and Testing:
Model training and classification consist of two stages. The first stage is the feature extraction stage. In this stage, feature extraction is performed using deep transfer learning architectures.
The second stage is the classification of the extracted features. The features are classified using classical machine learning techniques.

Evaluation Metrics
Confusion matrix is ​​one of the basic tools used to evaluate the performance of the model in classification problems. It visualizes the correct and incorrect predictions of the model. 
Accuracy: It is the ratio of the number of correctly predicted examples by the model to the total number of examples. It is an appropriate metric if all classes are balanced.
	
Precision: It shows how many of the samples that the model predicted as positive are actually positive. It is used when false positives are important.
	
Recall: It shows how many of the true positive examples were correctly predicted by the model. It is preferred in cases where missed positives are important.
	
F-score: It is the harmonic mean that balances between Precision and Recall. It is a more reliable metric, especially when the data is unbalanced.
	
Results:
The achieved accuracy, classification report, and confusion matrix for all the chosen models on test data is given and briefly discussed in the submitted manuscript. 
Limitations:
The generalizability of the model is affected by several limitations. First, the different standards of the images used may cause the features learned by the model during training to be inconsistent across different environments and devices. This may negatively affect the accuracy of the model unless it is tested on real-world data. In addition, the high GPU requirements of deep learning models may limit their widespread use in clinical settings, as such hardware may not always be available in all environments. When the model is trained on small and imbalanced datasets, it carries the risk of overfitting, which may cause the model to perform poorly on a larger dataset by only fitting the training data. Finally, the use of deep learning models in clinical settings may pose challenges in terms of security, interpretability, and compatibility.


Installation:
The necessary dependencies (libraries/ packages) associated with the ALL disease classification task were imported as follows:
import os
import time
import shutil
import pathlib
import itertools

# import data handling tools
import cv2
import numpy as np
import pandas as pd
import seaborn as sns
#sns.set_style('darkgrid')
import matplotlib.pyplot as plt
from sklearn.model_selection import train_test_split
from sklearn.metrics import confusion_matrix, classification_report

# import Deep learning Libraries
import tensorflow as tf
from tensorflow import keras
from tensorflow.keras.models import Sequential
from tensorflow.keras.optimizers import Adam, Adamax
from tensorflow.keras.metrics import categorical_crossentropy
from tensorflow.keras.preprocessing.image import ImageDataGenerator
from tensorflow.keras.layers import Conv2D, MaxPooling2D, Flatten, Dense, Activation, Dropout, BatchNormalization
from tensorflow.keras import regularizers

# Ignore Warnings
import warnings
warnings.filterwarnings("ignore")

print ('modules loaded')

Overview of the Functions Used in Preprocessing Steps
Various helper functions are utilized in preprocessing steps to make the input data compatible with the deep learning models. The description of the functions is given below:
o	make_dataframes(sdir): This function is use to load the data from source directory and further splits the data into three dataframes of training, validation and testing data for specified purposes.  The code of this helper function is given below:

def define_paths(dir):
    filepaths = []
    labels = []

    folds = os.listdir(dir)
    for fold in folds:
        foldpath = os.path.join(dir, fold)
        filelist = os.listdir(foldpath)

        for fold_ in filelist:
            foldpath_ = os.path.join(foldpath, fold_)
            filelist_ = os.listdir(foldpath_)

            for file_ in filelist_:
                fpath = os.path.join(foldpath_, file_)
                filepaths.append(fpath)
                labels.append(fold_)

    return filepaths, labels


# Concatenate data paths with labels into one dataframe ( to later be fitted into the model )
def define_df(files, classes):
    Fseries = pd.Series(files, name= 'filepaths')
    Lseries = pd.Series(classes, name='labels')
    return pd.concat([Fseries, Lseries], axis= 1)


# Function that create dataframe for train, validation, and test data
def create_df(data_dir):

    # train dataframe
    files, classes = define_paths(data_dir)
    df = define_df(files, classes)
    
    strat = df['labels']
    train_df, dummy_df = train_test_split(df, train_size=0.7, shuffle=True, random_state=123, stratify=strat)

    # test dataframe   
    strat = dummy_df['labels']
    valid_df, test_df= train_test_split(dummy_df, train_size=0.5, shuffle=True, random_state=123, stratify=strat)

    return train_df, valid_df, test_df

def create_model_data (train_df, valid_df, test_df, batch_size):
   


    # define model parameters
    img_size = (300, 300)
    channels = 3 # either BGR or Grayscale
    color = 'rgb'
    img_shape = (img_size[0], img_size[1], channels)

    # Recommended : use custom function for test data batch size, else we can use normal batch size.
    ts_length = len(test_df)
    test_batch_size = max(sorted([ts_length // n for n in range(1, ts_length + 1) if ts_length%n == 0 and ts_length/n <= 80]))
    test_steps = ts_length // test_batch_size

    # This function which will be used in image data generator for data augmentation, it just take the image and return it again.
    def scalar(img):
        return img

    tr_gen = ImageDataGenerator(preprocessing_function= scalar) #horizontal_flip= True
    ts_gen = ImageDataGenerator(preprocessing_function= scalar)

    train_gen = tr_gen.flow_from_dataframe( train_df, x_col= 'filepaths', y_col= 'labels', target_size= img_size, class_mode= 'categorical',
                                        color_mode= color, shuffle= False, batch_size= batch_size)

    valid_gen = ts_gen.flow_from_dataframe( valid_df, x_col= 'filepaths', y_col= 'labels', target_size= img_size, class_mode= 'categorical',
                                        color_mode= color, shuffle= False, batch_size= batch_size)

    # Note: we will use custom test_batch_size, and make shuffle= false
    test_gen = ts_gen.flow_from_dataframe( test_df, x_col= 'filepaths', y_col= 'labels', target_size= img_size, class_mode= 'categorical',
                                        color_mode= color, shuffle= False, batch_size= test_batch_size)

    return train_gen, valid_gen, test_gen
Visualize results and print values ​​to the screen

def show_images(gen):
    '''
    This function take the data generator and show sample of the images
    '''

    # return classes , images to be displayed
    g_dict = gen.class_indices        # defines dictionary {'class': index}
    classes = list(g_dict.keys())     # defines list of dictionary's kays (classes), classes names : string
    images, labels = next(gen)        # get a batch size samples from the generator

    # calculate number of displayed samples
    length = len(labels)        # length of batch size
    sample = min(length, 25)    # check if sample less than 25 images

    plt.figure(figsize= (20, 20))

    for i in range(sample):
        plt.subplot(5, 5, i + 1)
        image = images[i] / 255       # scales data to range (0 - 255)
        plt.imshow(image)
        index = np.argmax(labels[i])  # get image index
        class_name = classes[index]   # get class of image
        plt.title(class_name, color= 'blue', fontsize= 12)
        plt.axis('off')
    plt.show()
    
    
 def plot_label_count(df, plot_title):
    '''
    This function take df and plot labels value counts
    '''

    # Define needed variables
    vcounts = df['labels'].value_counts()
    labels = vcounts.keys().tolist()
    values = vcounts.tolist()
    lcount = len(labels)

    if lcount > 55:
        print('The number of labels is > 55, no plot will be produced')

    else:
        plot_labels(lcount, labels, values, plot_title)

def plot_labels(lcount, labels, values, plot_title):
    width = lcount * 4
    width = np.min([width, 20])

    plt.figure(figsize= (width, 5))

    form = {'family': 'serif', 'color': 'blue', 'size': 25}
    sns.barplot(labels, values)
    plt.title(f'Images per Label in {plot_title} data', fontsize= 24, color= 'blue')
    plt.xticks(rotation= 90, fontsize= 18)
    plt.yticks(fontsize= 18)
    plt.xlabel('CLASS', fontdict= form)
    yaxis_label = 'IMAGE COUNT'
    plt.ylabel(yaxis_label, fontdict= form)

    rotation = 'vertical' if lcount >= 8 else 'horizontal'
    for i in range(lcount):
        plt.text(i, values[i] / 2, str(values[i]), fontsize= 12,
                rotation= rotation, color= 'yellow', ha= 'center')
   plt.show()
·	Plotting confusion matrix and loss graphs

def plot_training(hist):
    '''
    This function take training model and plot history of accuracy and losses with the best epoch in both of them.
    '''

    # Define needed variables
    tr_acc = hist.history['accuracy']
    tr_loss = hist.history['loss']
    val_acc = hist.history['val_accuracy']
    val_loss = hist.history['val_loss']
    index_loss = np.argmin(val_loss)
    val_lowest = val_loss[index_loss]
    index_acc = np.argmax(val_acc)
    acc_highest = val_acc[index_acc]
    Epochs = [i+1 for i in range(len(tr_acc))]
    loss_label = f'best epoch= {str(index_loss + 1)}'
    acc_label = f'best epoch= {str(index_acc + 1)}'

    # Plot training history
    plt.figure(figsize= (20, 8))
    plt.style.use('fivethirtyeight')
    #plt.style.use('classic')
    plt.subplot(1, 2, 1)
    plt.plot(Epochs, tr_loss, 'r', label= 'Training loss')
    plt.plot(Epochs, val_loss, 'g', label= 'Validation loss')
    plt.scatter(index_loss + 1, val_lowest, s= 150, c= 'blue', label= loss_label)
    plt.title('Training and Validation Loss')
    plt.xlabel('Epochs')
    plt.ylabel('Loss')
    plt.legend()

    plt.subplot(1, 2, 2)
    plt.plot(Epochs, tr_acc, 'r', label= 'Training Accuracy')
    plt.plot(Epochs, val_acc, 'g', label= 'Validation Accuracy')
    plt.scatter(index_acc + 1 , acc_highest,s= 150, c= 'blue', label= acc_label)
    plt.title('Training and Validation Accuracy')
    plt.xlabel('Epochs')
    plt.ylabel('Accuracy')
    plt.legend()

    plt.tight_layout
    plt.show()
    
    
def plot_confusion_matrix(cm, classes, normalize= False, title= 'Confusion Matrix', cmap= plt.cm.Blues):
	'''
	This function plot confusion matrix method from sklearn package.
	'''

	plt.figure(figsize= (10, 10))
	plt.imshow(cm, interpolation= 'nearest', cmap= cmap)
	plt.title(title)
	plt.colorbar()

	tick_marks = np.arange(len(classes))
	plt.xticks(tick_marks, classes, rotation= 45)
	plt.yticks(tick_marks, classes)

	if normalize:
		cm = cm.astype('float') / cm.sum(axis= 1)[:, np.newaxis]
		print('Normalized Confusion Matrix')

	else:
		print('Confusion Matrix, Without Normalization')

	print(cm)

	thresh = cm.max() / 2.
	for i, j in itertools.product(range(cm.shape[0]), range(cm.shape[1])):
		plt.text(j, i, cm[i, j], horizontalalignment= 'center', color= 'white' if cm[i, j] > thresh else 'black')

	plt.tight_layout()
	plt.ylabel('True Label')
	plt.xlabel('Predicted Label')


·	(batch_size, train_df, test_df, valid_df, img_size): ImageDataGenerator class is exploited to create training, validation and testing sets. Following parameters are specified for each set:

data_dir = '/kaggle/input/alljpg10kkirpilmis' 

try:
    # Get splitted data
    train_df, valid_df, test_df = create_df(data_dir)

    # Get Generators
    batch_size = 32
    train_gen, valid_gen, test_gen = create_model_data(train_df, valid_df, test_df, batch_size)

except:
    print('Invalid Input')
    
# Create Model Structure
img_size = (224, 224)
channels = 3
img_shape = (img_size[0], img_size[1], channels)
class_count = len(list(train_gen.class_indices.keys())) # to define number of classes in dense layer
Callbacks:
The customized LR_ask() function is explored as a callback to automatically adjusts the learning rate by monitoring the performance of the model during training which gives valuable insights on the models learning behavior. 
class MyCallback(keras.callbacks.Callback):
    def __init__(self, model, patience, stop_patience, threshold, factor, batches, epochs, ask_epoch):
        super(MyCallback, self).__init__()
        self.model = model
        self.patience = patience # specifies how many epochs without improvement before learning rate is adjusted
        self.stop_patience = stop_patience # specifies how many times to adjust lr without improvement to stop training
        self.threshold = threshold # specifies training accuracy threshold when lr will be adjusted based on validation loss
        self.factor = factor # factor by which to reduce the learning rate
        self.batches = batches # number of training batch to run per epoch
        self.epochs = epochs
        self.ask_epoch = ask_epoch
        self.ask_epoch_initial = ask_epoch # save this value to restore if restarting training

        # callback variables
        self.count = 0 # how many times lr has been reduced without improvement
        self.stop_count = 0
        self.best_epoch = 1   # epoch with the lowest loss
        self.initial_lr = float(tf.keras.backend.get_value(model.optimizer.lr)) # get the initial learning rate and save it
        self.highest_tracc = 0.0 # set highest training accuracy to 0 initially
        self.lowest_vloss = np.inf # set lowest validation loss to infinity initially
        self.best_weights = self.model.get_weights() # set best weights to model's initial weights
        self.initial_weights = self.model.get_weights()   # save initial weights if they have to get restored

    # Define a function that will run when train begins
    def on_train_begin(self, logs= None):
        msg = 'Do you want model asks you to halt the training [y/n] ?'
        print(msg)
        ans = input('')
        if ans in ['Y', 'y']:
            self.ask_permission = 1
        elif ans in ['N', 'n']:
            self.ask_permission = 0

        msg = '{0:^8s}{1:^10s}{2:^9s}{3:^9s}{4:^9s}{5:^9s}{6:^9s}{7:^10s}{8:10s}{9:^8s}'.format('Epoch', 'Loss', 'Accuracy', 'V_loss', 'V_acc', 'LR', 'Next LR', 'Monitor','% Improv', 'Duration')
        print(msg)
        self.start_time = time.time()


    def on_train_end(self, logs= None):
        stop_time = time.time()
        tr_duration = stop_time - self.start_time
        hours = tr_duration // 3600
        minutes = (tr_duration - (hours * 3600)) // 60
        seconds = tr_duration - ((hours * 3600) + (minutes * 60))

        msg = f'training elapsed time was {str(hours)} hours, {minutes:4.1f} minutes, {seconds:4.2f} seconds)'
        print(msg)

        # set the weights of the model to the best weights
        self.model.set_weights(self.best_weights)


    def on_train_batch_end(self, batch, logs= None):
        # get batch accuracy and loss
        acc = logs.get('accuracy') * 100
        loss = logs.get('loss')

        # prints over on the same line to show running batch count
        msg = '{0:20s}processing batch {1:} of {2:5s}-   accuracy=  {3:5.3f}   -   loss: {4:8.5f}'.format(' ', str(batch), str(self.batches), acc, loss)
        print(msg, '\r', end= '')


    def on_epoch_begin(self, epoch, logs= None):
        self.ep_start = time.time()


    # Define method runs on the end of each epoch
    def on_epoch_end(self, epoch, logs= None):
        ep_end = time.time()
        duration = ep_end - self.ep_start

        lr = float(tf.keras.backend.get_value(self.model.optimizer.lr)) # get the current learning rate
        current_lr = lr
        acc = logs.get('accuracy')  # get training accuracy
        v_acc = logs.get('val_accuracy')  # get validation accuracy
        loss = logs.get('loss')  # get training loss for this epoch
        v_loss = logs.get('val_loss')  # get the validation loss for this epoch

        if acc < self.threshold: # if training accuracy is below threshold adjust lr based on training accuracy
            monitor = 'accuracy'
            if epoch == 0:
                pimprov = 0.0
            else:
                pimprov = (acc - self.highest_tracc ) * 100 / self.highest_tracc # define improvement of model progres

            if acc > self.highest_tracc: # training accuracy improved in the epoch
                self.highest_tracc = acc # set new highest training accuracy
                self.best_weights = self.model.get_weights() # training accuracy improved so save the weights
                self.count = 0 # set count to 0 since training accuracy improved
                self.stop_count = 0 # set stop counter to 0
                if v_loss < self.lowest_vloss:
                    self.lowest_vloss = v_loss
                self.best_epoch = epoch + 1  # set the value of best epoch for this epoch

            else:
                # training accuracy did not improve check if this has happened for patience number of epochs
                # if so adjust learning rate
                if self.count >= self.patience - 1: # lr should be adjusted
                    lr = lr * self.factor # adjust the learning by factor
                    tf.keras.backend.set_value(self.model.optimizer.lr, lr) # set the learning rate in the optimizer
                    self.count = 0 # reset the count to 0
                    self.stop_count = self.stop_count + 1 # count the number of consecutive lr adjustments
                    self.count = 0 # reset counter
                    if v_loss < self.lowest_vloss:
                        self.lowest_vloss = v_loss
                else:
                    self.count = self.count + 1 # increment patience counter

        else: # training accuracy is above threshold so adjust learning rate based on validation loss
            monitor = 'val_loss'
            if epoch == 0:
                pimprov = 0.0

            else:
                pimprov = (self.lowest_vloss - v_loss ) * 100 / self.lowest_vloss

            if v_loss < self.lowest_vloss: # check if the validation loss improved
                self.lowest_vloss = v_loss # replace lowest validation loss with new validation loss
                self.best_weights = self.model.get_weights() # validation loss improved so save the weights
                self.count = 0 # reset count since validation loss improved
                self.stop_count = 0
                self.best_epoch = epoch + 1 # set the value of the best epoch to this epoch

            else: # validation loss did not improve
                if self.count >= self.patience - 1: # need to adjust lr
                    lr = lr * self.factor # adjust the learning rate
                    self.stop_count = self.stop_count + 1 # increment stop counter because lr was adjusted
                    self.count = 0 # reset counter
                    tf.keras.backend.set_value(self.model.optimizer.lr, lr) # set the learning rate in the optimizer

                else:
                    self.count = self.count + 1 # increment the patience counter

                if acc > self.highest_tracc:
                    self.highest_tracc = acc

        msg = f'{str(epoch + 1):^3s}/{str(self.epochs):4s} {loss:^9.3f}{acc * 100:^9.3f}{v_loss:^9.5f}{v_acc * 100:^9.3f}{current_lr:^9.5f}{lr:^9.5f}{monitor:^11s}{pimprov:^10.2f}{duration:^8.2f}'
        print(msg)

        if self.stop_count > self.stop_patience - 1: # check if learning rate has been adjusted stop_count times with no improvement
            msg = f' training has been halted at epoch {epoch + 1} after {self.stop_patience} adjustments of learning rate with no improvement'
            print(msg)
            self.model.stop_training = True # stop training

        else:
            if self.ask_epoch != None and self.ask_permission != 0:
                if epoch + 1 >= self.ask_epoch:
                    msg = 'enter H to halt training or an integer for number of epochs to run then ask again'
                    print(msg)

                    ans = input('')
                    if ans == 'H' or ans == 'h':
                        msg = f'training has been halted at epoch {epoch + 1} due to user input'
                        print(msg)
                        self.model.stop_training = True # stop training

                    else:
                        try:
                            ans = int(ans)
                            self.ask_epoch += ans
                            msg = f' training will continue until epoch {str(self.ask_epoch)}'
                            print(msg)
                            msg = '{0:^8s}{1:^10s}{2:^9s}{3:^9s}{4:^9s}{5:^9s}{6:^9s}{7:^10s}{8:10s}{9:^8s}'.format('Epoch', 'Loss', 'Accuracy', 'V_loss', 'V_acc', 'LR', 'Next LR', 'Monitor', '% Improv', 'Duration')
                            print(msg)

                        except Exception:
                            print('Invalid')


Network Architecture:
base_model = tf.keras.applications.Xception(include_top= False, weights= "imagenet", input_shape= img_shape, pooling= 'max')

model = Sequential([
    base_model,
    BatchNormalization(axis= -1, momentum= 0.99, epsilon= 0.001),
    Dense(256, kernel_regularizer= regularizers.l2(l= 0.016), activity_regularizer= regularizers.l1(0.006),
                bias_regularizer= regularizers.l1(0.006), activation= 'relu'),
    Dropout(rate= 0.45, seed= 123),
    Dense(class_count, activation= 'softmax')
])

model.compile(Adamax(learning_rate= 0.001), loss= 'categorical_crossentropy', metrics= ['accuracy'])

model.summary()
·	Hyperparameter settings and model training
batch_size = 32   # set batch size for training
epochs = 20   # number of all epochs in training
patience = 1   #number of epochs to wait to adjust lr if monitored value does not improve
stop_patience = 10   # number of epochs to wait before stopping training if monitored value does not improve
threshold = 0.9   # if train accuracy is < threshold adjust monitor accuracy, else monitor validation loss
factor = 0.5   # factor to reduce lr by
ask_epoch = 5   # number of epochs to run before asking if you want to halt training
batches = int(np.ceil(len(train_gen.labels) / batch_size))    # number of training batch to run per epoch

callbacks = [MyCallback(model= model, patience= patience, stop_patience= stop_patience, threshold= threshold,
            factor= factor, batches= batches, epochs= epochs, ask_epoch= ask_epoch )]

history = model.fit(x= train_gen, epochs= epochs, verbose= 0, callbacks= callbacks,
                    validation_data= valid_gen, validation_steps= None, shuffle= False)

Evaluating Models Performance:
The performance of the models is validated through their predictions on unseen test data. The confusion matrix and classification report are computed to visualize the number of correct and misclassified instances.
preds=model.predict(test_gen)  

test_label=test_gen.labels
pred=np.argmax(preds,axis=1)
from sklearn.metrics import classification_report

print(classification_report(test_label,pred,digits=4))

names =[ "NORMAL", "ALL"]
from sklearn.metrics import confusion_matrix
from sklearn.metrics import f1_score,precision_score,recall_score,accuracy_score
from sklearn.model_selection import GridSearchCV
fig = plt.figure(figsize=(5, 5), dpi=100)
cm = confusion_matrix(test_label, pred)
sns.heatmap(cm,vmin=0, fmt='g', annot=True, cbar=False,cmap=plt.cm.Blues, xticklabels=names, yticklabels=names)

plt.xlabel('Predicted label')
plt.ylabel('True label')
plt.title('Confusion Matrix')

import math
from sklearn.metrics import roc_curve, auc
import matplotlib.pyplot as plt
import matplotlib.patches as patches
import random

test_label=np.array(test_label)

probs = np.exp(preds[:,1])
fpr, tpr, thresholds = roc_curve(test_label, probs, pos_label=1)
roc_auc = auc(fpr, tpr)
print('ROC area is {0}'.format(roc_auc))

plt.figure(dpi=100)
plt.plot(fpr, tpr, color='darkorange', label='ROC curve (area = %0.2f)' % roc_auc)
plt.plot([0, 1], [0, 1], color='navy', linestyle='--')
plt.xlim([-0.01, 1.0])
plt.ylim([0.0, 1.01])
plt.xlabel('False Positive Rate')
plt.ylabel('True Positive Rate')
plt.title('Receiver operating characteristic')
plt.legend(loc="lower right")

Feature Extraction :
model_feat = Model(inputs=model.inputs,outputs=model.get_layer('batch_normalization_4').output)
feat_train = model_feat.predict(train_gen)
print(feat_train.shape)
labels=train_gen.labels
train_label=np.array(labels)
print(train_label.shape)

feat_test = model_feat.predict(test_gen)
print(feat_test.shape)
test_label=test_gen.labels
test_label=np.array(test_label)
print(test_label.shape)

feat_valid = model_feat.predict(valid_gen)
print(feat_valid.shape)
valid_label=valid_gen.labels
valid_label=np.array(valid_label)
print(valid_label.shape)


feat_train=pd.DataFrame(feat_train)

train_label=pd.DataFrame(train_label)

feat_valid=pd.DataFrame(feat_valid)

valid_label=pd.DataFrame(valid_label)

#x_train_valid=pd.concat([feat_train, feat_valid], axis=0)

#y_train_valid=pd.concat([train_label, valid_label], axis=0)


feat_test=pd.DataFrame(feat_test)

test_label=pd.DataFrame(test_label)

x_train_valid=pd.concat([feat_train, feat_valid,feat_test], axis=0)

y_train_valid=pd.concat([train_label, valid_label,test_label], axis=0)

from sklearn.model_selection import train_test_split
x_train, x_test, y_train, y_test = train_test_split(x_train_valid, y_train_valid, test_size = 0.15, random_state = 2)


f_train=np.array(feat_train)
f_test=np.array(feat_test)
f_valid=np.array(feat_valid)

·	Classification of extracted features: The algorithm to be used instead of random forest can be added in the classification phase.
from sklearn.model_selection import cross_val_score,cross_val_predict
from sklearn.ensemble import RandomForestClassifier
from sklearn.naive_bayes import GaussianNB
from sklearn.neighbors import KNeighborsClassifier
from sklearn import svm
from sklearn.metrics import confusion_matrix
from sklearn.metrics import f1_score,precision_score,recall_score,accuracy_score
from sklearn.model_selection import GridSearchCV

r_forest = RandomForestClassifier(200,max_depth=5, random_state=2)
r_forest.fit(x_train,y_train)
predicted = r_forest.predict(x_test)
score = r_forest.score(x_test, y_test)
rf_score_ = np.mean(score)

print('Accuracy : %.5f' % (rf_score_))

print("Classification Report:")
print(classification_report(y_test, predicted,digits=4))

# Confusion matrix
names =[ "NORMAL", "ALL"]
from sklearn.metrics import confusion_matrix
from sklearn.metrics import f1_score,precision_score,recall_score,accuracy_score
from sklearn.model_selection import GridSearchCV
fig = plt.figure(figsize=(5, 5), dpi=100)
cm = confusion_matrix(y_test, predicted)
sns.heatmap(cm,vmin=0, fmt='g', annot=True, cbar=False,cmap=plt.cm.Blues, xticklabels=names, yticklabels=names)

plt.xlabel('Predicted label')
plt.ylabel('True label')
plt.title('Confusion Matrix')

# ROC eðrisi çizme
# Not: ROC eðrisi ve AUC, yalnýzca ikili sýnýflandýrma problemleri için anlamlýdýr.
if len(np.unique(y_train)) == 2:
    y_prob = r_forest.predict_proba(x_test)[:, 1]
    fpr, tpr, _ = roc_curve(y_test, y_prob)
    roc_auc = auc(fpr, tpr)
    
    plt.figure()
    plt.plot(fpr, tpr, color='darkorange', lw=2, label='ROC curve (area = %0.2f)' % roc_auc)
    plt.plot([0, 1], [0, 1], color='navy', lw=2, linestyle='--')
    plt.xlim([0.0, 1.0])
    plt.ylim([0.0, 1.05])
    plt.xlabel('False Positive Rate')
    plt.ylabel('True Positive Rate')
    plt.title('Receiver Operating Characteristic (ROC)')
    plt.legend(loc="lower right")
    plt.show()
else:
    print("ROC curve is only meaningful for binary classification.")
    
# Other machine learning algorithms
